# Medication-Related Osteonecrosis of the Jaws: A Comparison of SICMF–SIPMO and AAOMS Guidelines

**DOI:** 10.3390/diagnostics13132137

**Published:** 2023-06-21

**Authors:** Davide De Cicco, Ciro Emiliano Boschetti, Mario Santagata, Giuseppe Colella, Samuel Staglianò, Alexander Gaggl, Gian Battista Bottini, Rita Vitagliano, Salvatore D’amato

**Affiliations:** 1 Department of Neurosciences, Reproductive and Odontostomatological Sciences, University of Naples “Federico II”, 80131 Naples, Italy; davide.decicco@unina.it; 2 Department of Multidisciplinary Medical, Surgical and Dental Specialties, University of Campania “Luigi Vanvitelli”, 80138 Naples, Italy; ciroemiliano.boschetti@unicampania.it (C.E.B.); mario.santagata@unicampania.it (M.S.); giuseppe.colella@unicampania.it (G.C.); samuel.stagliano@studenti.unicampania.it (S.S.); salvatore.damato@unicampania.it (S.D.); 3 Department of Oral and Craniomaxillofacial Surgery, Paracelsus Medical University, 5020 Salzburg, Austria; a.gaggl@salk.at (A.G.); g.bottini@salk.at (G.B.B.)

**Keywords:** MRONJ, ONJ, guidelines, diagnosis, treatment, prevention

## Abstract

(1) Background: Medication-related osteonecrosis of the jaws (MRONJ) is an adverse drug reaction characterized by progressive bone disruption and necrosis in the mandibular and/or maxillary bones. It occurs in individuals who have received antiresorptive drugs without prior radiotherapy. Since its first reported cases in the USA in 2003, extensive literature has emerged worldwide, leading to significant advancements in understanding MRONJ’s pathogenesis and management. (2) Results: This article aims to compare the current national recommendations provided by the Italian Society of Maxillofacial Surgery (SICMF)/Italian Society of Oral Pathology and Medicine (SIPMO) and the American Association of Oral and Maxillofacial Surgeons (AAOMS). (3) Conclusions: Historically, the AAOMS advocated for a more conservative approach compared to the Italian guidelines. However, in their 2022 update, the AAOMS adopted a different perspective based on reported evidence, highlighting the advantages of early surgical treatment. Despite resolving some initial controversies, differences still exist between the two sets of recommendations, particularly regarding diagnosis and staging.

## 1. Introduction

Medication-related osteonecrosis of the jaws (MRONJ) is an unfortunate adverse event observed in patients who receive bone antiresorptive drugs (bone-modifying agents—BMA) or certain monoclonal antibodies. This disease consists of the necrosis of one or both jaws, either partially or completely, and has a multifactorial etiology. The classic presentation typically includes bone exposure through the oral mucosa, accompanied by varying degrees of local infection. It is crucial to differentiate MRONJ from osteoradionecrosis of the jaws (ORNJ), which specifically pertains to patients who have undergone radiotherapy in the head and neck region.

Affected patients may present a wide spectrum of disease severity, ranging from completely asymptomatic individuals to those with severe limitations in their performance status. In such cases, MRONJ has been shown to significantly impair patients’ quality of life, exacerbating an already compromised clinical condition. The drugs associated with a risk of MRONJ complications are predominantly administered to oncological patients, who commonly experience advanced disease stages and undergo various therapies to treat it. Considering these factors allows physicians to approach MRONJ from the most effective holistic perspective and define an optimal treatment strategy.

### 1.1. MRONJ History

MRONJ is a relatively recent pathology that was first described in 2003 by Wang et al. in San Francisco, CA, USA [1]. The authors reported cases of avascular bone necrosis affecting the jaws in three women who received adjuvant chemotherapies for metastatic breast cancer. Among the chemotherapeutic agents used, pamidronate was administered to counteract skeletal metastases. Two patients underwent dental extractions during active treatment, resulting in socket degeneration and exposure of necrotic bone. The third patient developed an oro-antral fistula spontaneously. Due to the absence of a scientifically proven correlation between pamidronate and MRONJ, the authors referred to these events as “chemotherapy-related osteonecrosis.” Subsequently, numerous reports on this condition emerged in the international literature until 2007, when the American Association of Oral and Maxillofacial Surgeons (AAOMS) proposed the first position paper on bisphosphonate-related osteonecrosis of the jaws (BRONJ) [2]. This seminal article reviewed the initial epidemiological data, established diagnostic criteria, and provided treatment guidelines. Subsequent updates were published in 2009 [3], 2014 [4], and 2022 [5], reflecting advancements in research. In 2019, a joint committee consisting of the Multinational Association of Supportive Care in Cancer (MASCC), the International Society of Oral Oncology (ISOO), and the American Society of Clinical Oncology (ASCO) conducted a systematic review of the literature to release a comprehensive practice guideline [6]. Notably, this review classified the findings based on the quality of evidence, yielding a comprehensive list of recommendations with varying levels of strength. The growing interest in establishing a unified protocol for MRONJ diagnosis and management is evident from the multitude of publications that have emerged worldwide over the past two decades. An important contribution was made in 2020 by the joint committee of the Italian Society of Maxillofacial Surgery (SICMF) and the Italian Society of Oral Pathology and Medicine (SIPMO) [7]. This Italian expert board introduced a significant innovation in the diagnostic workflow, incorporating radiological criteria based on CT scans.

### 1.2. Definition

According to the SICMF–SIPMO guidelines [7], MRONJ is defined as an “adverse drug reaction characterized by progressive disruption and necrosis of the mandibular and/or maxillary bones, occurring in subjects who received antiresorptive drugs without previous radiotherapy”. Therefore, MRONJ can develop not only in oncological patients but also in individuals receiving such therapies, including those with osteoporosis. This fact has significant implications for managing the resulting complications, as discussed below.

In contrast, the 2022 update from the American Association of Oral and Maxillofacial Surgeons (AAOMS) provided a more clinical definition based on the presence of the following features [5]:

1. Current or previous treatment with antiresorptive therapy alone or in combination with immune modulators or antiangiogenic medications.
2. Exposed bone or bone that can be probed through an intraoral or extraoral fistula(e) in the maxillofacial region that has persisted for more than eight weeks.
3. No history of radiation therapy to the jaws or metastatic disease in the jaws.

Over the years, various definitions and taxonomic denominations have been used to refer to what is now comprehensively included under the term MRONJ. While this discussion is beyond the scope of this article, it is important for practitioners to be aware that osteonecrosis of the jaws may develop as a consequence of various medications, not only bisphosphonates as previously believed.

### 1.3. Etiology and Pathogenesis

MRONJ can be classified based on the type of medication that could have triggered this adverse event. Two fundamental categories can be recognized: those associated with bisphosphonates (bisphosphonate-related osteonecrosis of the jaws—BRONJ) and those associated with other medications (non-BRONJ) (Table 1). This distinction is due to the diffusion of modern monoclonal antibody therapies. Post-market trials have clearly demonstrated an association between MRONJ and monoclonal antibodies, particularly for those medications targeting the receptor activator of nuclear factor kappa-Β ligand (RANKL), such as denosumab [8]. Denosumab is the only monoclonal antibody included in the group of BMAs, as well as bisphosphonates, and is considered responsible for non-BRONJ disease. Other drugs that have shown a role in triggering non-BRONJ include inhibitors of the vascular endothelial growth factor receptor (anti-VEGFR), tyrosine kinase inhibitors, and mammalian target of rapamycin inhibitors (anti-mTOR).

The pathogenesis of MRONJ remains a debated and incompletely understood process. Various risk factors have been identified as contributors to disease development, including diabetes, smoking, the use of removable dental prostheses, poor oral hygiene, and recent history of invasive dental procedures [6]. Understanding the basic physiology of bone tissue is essential to comprehend how and where MRONJ initiates.

In healthy bone, osteocytes undergo cellular senescence and complete their life cycle through apoptotic or autophagic processes. The precise mechanism is not fully understood, but the resulting apoptotic bodies are eliminated through phagocytosis and tissue clearance, facilitated by osteoclasts and osteoblasts during bone turnover. Normally, this process occurs without eliciting an inflammatory response from the immune system, in contrast to cellular necrosis. However, if there is a delay in removing the apoptotic bodies, they may undergo secondary necrosis and inflammation. Considering that bone-modifying agents (BMAs) act by suppressing bone turnover and reducing osteoclast activity, apoptotic bodies may remain within the surrounding bone for an extended period. This mechanism is believed to increase the risk of secondary necrosis and the development of the inflammatory basis of MRONJ disease. The inflammatory response is sustained by pro-inflammatory cytokines, such as TNF-α and IL-1, which also contribute to the necrosis of other osteocytes within the surrounding bone. This necrosis prompts the formation of a vicious circle, intensifying inflammation and the death of additional osteocytes, thereby exacerbating the spread of the disease [8].

Currently, it is not clearly understood how the process limits its expansion in cases where the disease involves only a portion of the bony structure. However, according to the published literature, it is evident that different factors must coexist to promote the development of the disease. These factors are classified as predisposing factors (e.g., BMAs assumption) and risk factors within the oral cavity (e.g., periodontic disease, periapical lesions or inflammations, or inflammatory response to dental extractions).

Aguirre et al. have recently published a comprehensive review that is highly recommended for gaining a thorough understanding of the biological basis of MRONJ [8].

## 2. Material and Methods

**Figure 1:**
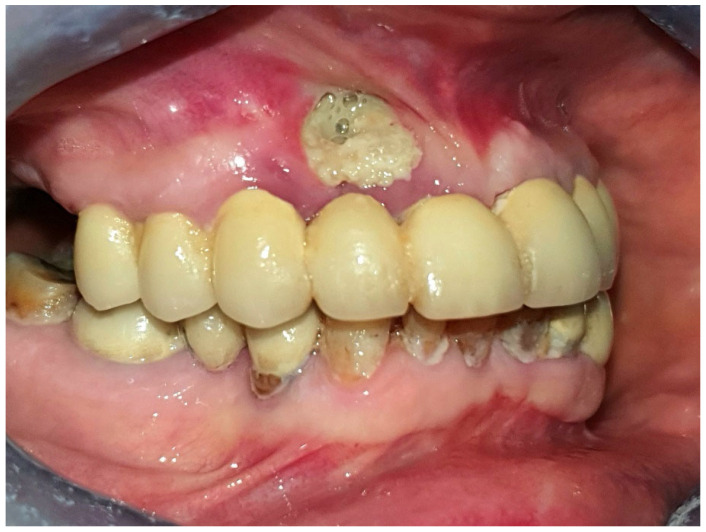
Visible necrotic bone is observed in the buccal region of the maxilla, specifically in the area of the upper right lateral incisor. This presentation is considered a typical manifestation of MRONJ. It is important to note the compromised dental hygiene status and the widespread lesions affecting the lower denture, both of which serve as red flags indicating a predisposition to develop MRONJ.

The most typical presentation of MRONJ is when patients are referred to the specialist due to the development of exposed necrotic bone through otherwise healthy mucosa. This development is often accompanied by pain and suppuration in the affected region (Figure 1). Diagnosing such clear cases is relatively easy and straightforward, but in some patients, the clinical presentation may not be as obvious. Timely recognition and treatment of MRONJ can have a positive impact on the patient’s functional prognosis and performance status.

### 2.1. Diagnosis and Staging—Case History

Obtaining a detailed patient history is crucial for establishing the background. It is important to identify the underlying pathology that necessitated treatment with BMAs or other MRONJ-related therapies. Additionally, any previous radiation to the head and neck region should be excluded. The specific therapy being administered, the duration of treatment, whether the patient is currently receiving treatment, and the route of administration (oral or intravenous) should all be determined. The onset and progression of symptoms should be carefully ascertained. The patient should be asked about any prior dental procedures, and any potential connection to the onset of symptoms should be clarified. In classic presentations, it is common to discover a history of dental extraction or other interventions performed during the course of therapy, followed by the development of initial symptoms. However, identifying the precise trigger for the disease may be challenging or even impossible in certain cases.

MRONJ should always be suspected in patients undergoing MRONJ-related therapies who complain of jaw pain, even in cases where there are minimal or no apparent oral cavity alterations or history of dental interventions. In most cases, the initial symptom is jaw pain that cannot be attributed to other causes. This pain is typically described as dull, continuous, and exacerbated by chewing or wearing removable prostheses. Although pain is reported by the majority of affected patients, it may be completely absent, even in advanced stages.

### 2.2. Diagnosis and Staging—Examination

Facial inspection may reveal swelling caused by the underlying inflammation or fistulas that drain purulent fluids (in advanced stages). The oral cavity should be examined systematically:

- Assess the oral hygiene status.
- Outline the dental formula, paying close attention to radicular remnants, caries, dental fractures, erosion, abrasion, and abfractions.
- Assess and classify the periodontal condition and eventual gingival recesses.
- Identify intraoral fistulas, oral–nasal or oral–antral communications, spots of purulent fluid leakage, areas of exposed necrotic bone

It is strongly recommended to explore the oral cavity during palpation to identify any areas of referred pain, which may uncover hidden spots of purulent fluid discharge. Palpation should also focus on signs typical of advanced stages, such as bone sequestra or pathological fractures in the affected bone, especially in the mandible.

**Figure 2:**
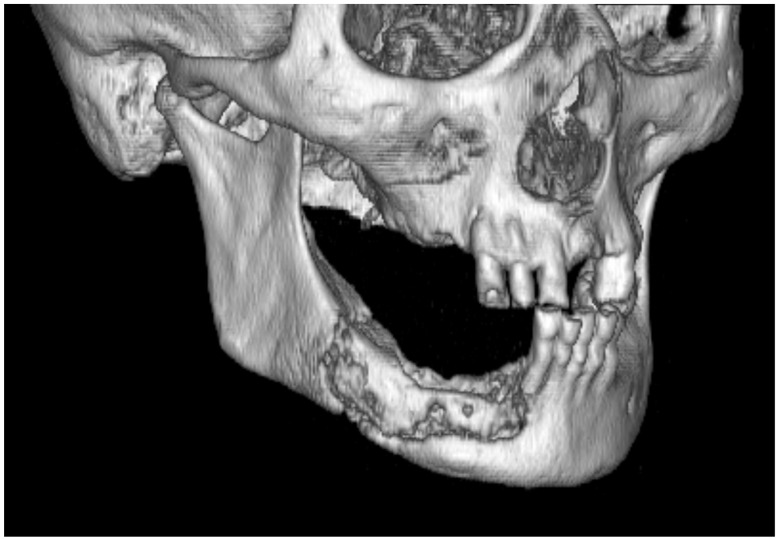
A bone sequestrum is observed in the right mandible, accompanied by a pathological fracture of the inferior border. Interestingly, this patient does not report pain but experiences abnormal mobility of the lower jaw, which hinders the use of the partial removable prosthesis in the lower right quadrant. During clinical examination, reciprocal abnormal movement of the mandibular stump and fluctuation of the bone sequestrum are noticeable.

In cases where bony exposure is present, the bone should be palpated to assess for mobility, which may indicate the presence of an underlying bone sequestrum (Figure 2). MRONJ can develop within a bone sequestrum, particularly if the disease has been present for a long period.

A dental examination is of paramount importance, particularly for patients who are about to undergo MRONJ-related therapies. Mobile dental elements should be identified and classified according to the Miller scale system (Table 2).

### 2.3. Diagnosis and Staging—Imaging

Radiological examination is essential for evaluating patients affected by or suspected of having MRONJ. Orthopantomography serves as the initial investigative method. It is mandatory for patients at risk of MRONJ to assess dental and periodontal health status, as well as the presence of osseous lesions (e.g., osteolytic radiolucencies). Obvious MRONJs are characterized by an irregular area of osteolysis, displaying a diffused radiolucency in the affected bone. These lesions typically arise within a sclerotic bone, which can sometimes make it challenging to determine the exact extent of the disease. In some cases, only mild rarefaction of the bony trabeculae may be present, which can be observed even in completely healthy patients but may also represent early stages of the disease. On the other hand, advanced stages of MRONJ may clearly exhibit signs of bone sequestra or pathological fractures.

**Figure 3:**
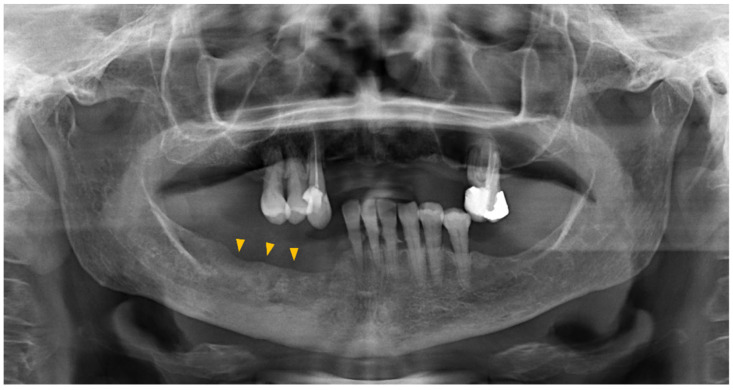
The orthopantomogram of the patient depicted in Figure 2 reveals osteosclerosis in the body of the right mandible, exhibiting minimal indications of bone resorption (indicated by yellow arrows) in comparison to the findings observed in the CT scan. It is worth mentioning that the CT scan also detected a fracture in the inferior border of the mandible, in contrast to what is observed in the orthopantomogram.

Orthopantomography has demonstrated limited sensitivity. Only cases in which bone resorption has resulted in a loss of at least 30–50% of mineral density can be clearly detected (Figure 3).

Therefore, certain cases may require a secondary-level examination to establish a diagnosis. The radiological findings obtained from a CT scan are typically comprehensive and serve as a conclusive component of the radiological assessment. The Italian 2020 guidelines introduced a classification system for radiological signs, categorizing them as early or advanced (Table 3).

Advanced investigations, such as MRI or PET, are reserved for uncertain cases or to differentiate MRONJ from other conditions (e.g., distant solid malignant bone metastases).

### 2.4. Staging

Several revisions have been reported since the first AAOMS 2009 staging proposal, reflecting scientific progress. However, controversies persist when comparing the main available staging systems. The AAOMS guidelines primarily stage MRONJ based on clinical presentation, while the SICMF–SIPMO guidelines incorporate detailed clinical and radiological criteria. The American board has been hesitant to include radiological findings as a diagnostic or staging criterion due to inconsistencies in clinical studies.

According to the current AAOMS classification (Table 4), MRONJ is categorized into four stages. In contrast, the SICMF–SIPMO 2020 recommendations outline three stages based on clinical and radiological findings (Table 4), omitting the stage 0 proposed by the American counterpart. Stage 0 refers to patients who exhibit suggestive symptoms without obvious radiological or clinical evidence of MRONJ. This classification rationale aligns with orthopedic practices, where stage 0 encompasses patients at risk of developing avascular necrosis without evident disease findings [5,9]. Both the Italian and American committees acknowledge that a significant percentage of patients with stage 0 MRONJ will progress to more severe stages. Based on this evidence, however, the SICMF–SIPMO board declined to include it in their proposed staging system [7,10].

## 3. Results and Discussion

### 3.1. MRONJ Management—Prevention

According to international recommendations, it is essential to assess and identify any associated risk factors in patients requiring MRONJ-related therapies. The responsibility lies with the specialist who evaluates the patient, as they need to recognize conditions that may potentially trigger the development of MRONJ. Therefore, establishing effective communication between the maxillofacial surgeon (or dentist) and the oncologist prior to initiating therapies is crucial. For patients already receiving MRONJ-related medications, they should be referred to a specialist for appropriate follow-up and to facilitate the reporting of any symptom onset.

The risk factors associated with MRONJ development can be categorized as follows:

- Drug-related risk factors
- Systemic risk factors
- Local risk factors

Drug-related risk factors are determined by the specific pharmacokinetic and pharmacodynamic properties of the medication being administered. These factors include:

- Drug class
- Bioavailability
- Administration route
- Cumulative drug dose, which is influenced by the drug half-life
- Duration of treatment

Bisphosphonates can be categorized into two groups: amino-bisphosphonates (aBP) and non-amino-bisphosphonates (naBP). The presence of two amino groups is a significant factor that distinguishes their affinity for bony hydroxyapatite [7]. This affinity affects the drug’s bioavailability, which remains notably high for an extended period, even after treatment cessation [11,12]. In MRONJ prevention, the goal is to maintain a stable clinical condition to minimize the risk of any further dentoalveolar interventions during or after antiresorptive treatment. Due to the substantial drug affinity, any additional intervention performed even months after treatment discontinuation may be considered at risk of MRONJ development.

The aBPs have been extensively studied, as they were the primary treatment choice for a considerable period. The introduction of anti-RANKL monoclonal antibodies (denosumab) has brought about a new approach to managing skeletal metastases from distant solid tumors. However, denosumab has shown a significantly higher risk of MRONJ development, despite having lower bioavailability and half-life compared to aBP [13].

Systemic risk factors include conditions that impact bone and drug metabolism, as well as oral health. Diabetes requires special attention due to its influence on bone and mucosal microcirculation. Prolonged exposure to high blood glucose levels has been observed to negatively affect bone tissue microarchitecture and healing through direct and indirect mechanisms [14]. Although an explicit role in the onset of MRONJ has not yet been established, uncontrolled diabetes may contribute to an unfavorable environment for its development [15].

Smoking has a certain detrimental effect on the development and progression of periodontal disease [16]. While the exact pathogenic basis remains unclear, various hypotheses propose that alterations in the microbiota and impaired immune response may play a role. Conversely, it has been observed that tobacco cessation significantly reduces the incidence of periodontal disease and is associated with its resolution when combined with periodontal treatments [16]. Therefore, patients receiving MRONJ-related medications should be adequately informed and encouraged to quit smoking.

All current literature guidelines concur on the importance of controlling local risk factors to prevent the onset of MRONJ. As a general principle, any condition that can promote inflammation or infection in the dental supporting structures should be considered a local risk factor. It is crucial for specialists to promptly recognize and treat these factors to ensure the safe initiation of medical therapy. The dental status should be thoroughly assessed to determine the most accurate dental prognosis, although a definitive consensus on this matter has not yet been reached [17]. Adequate treatment and resolution of deep pockets, dental calculus, gingival recessions, alveolar bone resorption with root exposure, dental caries, mobile teeth, and periapical lesions identified on radiological exams are necessary. Patients should be referred to an oral hygienist for professional treatment and to learn effective daily self-hygiene practices. This conservative approach can promote the resolution of even severe dental mobility and deep probing pockets, providing sufficient stability for safe drug administration. For cases of refractory periodontal diseases, minimally invasive periodontal interventions or dental splinting may be beneficial. Finally, teeth with a poor prognosis may require extraction or various surgical interventions if periapical lesions are detected on X-rays.

All surgical wounds, including incisions or residual dental sockets, must be fully healed before the patient undergoes anti-resorptive therapies initiated by the oncologist. The minimum healing time lacks a general consensus due to subjective variability and the extent of the intervention performed. It is the responsibility of the specialist to determine when the healing process has sufficiently progressed to refer the patient to the oncologist.

Maintaining optimal oral health is crucial throughout the therapeutic process. Therefore, periodic reassessment by the specialist and referral to a professional oral hygienist are necessary, aligning with the recommendations of both the American and Italian guidelines. However, in contrast to the AAOMS and MASCC–ISCOO–ASCO guidelines, the SICMF–SIPMO guidelines propose specific timing for patient evaluation and hygiene, recommending assessments every four months.

### 3.2. MRONJ Treatment

Over the past two decades, significant changes have occurred in the recommended management of MRONJ. Evolving scientific evidence has led to the refinement of treatment approaches to better align with the primary goals of MRONJ management: disease resolution and improved quality of life. Initially, in line with the first AAOMS recommendations, the emphasis in MRONJ treatment was on conservative measures and symptom resolution, avoiding invasive interventions in already compromised patients. However, recent literature has challenged this approach, demonstrating the advantages of surgical interventions even in select initial stages of MRONJ.

The treatment of MRONJ can be broadly classified into two main approaches: conservative (non-operative) and surgical.

#### 3.2.1. Conservative Treatment

The fundamental principle of conservative management in MRONJ is to address the underlying bacterial and infectious environment through the use of broad-spectrum antibiotics [7]. Infections are common complications of MRONJ and can potentially contribute to its development. Therefore, the administration of antibiotics is warranted for etiological reasons, but there is still a lack of conclusive evidence regarding the optimal choice of antibiotic and treatment plan in terms of dosage and duration.

The mainstay of antibiotic management for MRONJ involves a combination of amoxicillin/clavulanic acid (effective against Gram-negative and β-lactamase resistant Gram-positive bacteria) and metronidazole (effective against anaerobic and Gram-positive cocci), along with the use of oral antiseptic solutions (such as rinses with 0.2% chlorhexidine). The SICMF–SIPMO guidelines recommend limiting the use of antibiotics to acute infections and their relapses, while avoiding prolonged exposure to the same antibiotic. The recommended duration of therapy is at least seven days but should not exceed 21 days to minimize the risk of selecting resistant organisms [7].

The effectiveness of antibiotic therapy is still a subject of debate due to the limited level of evidence. Although rare, early stages of MRONJ may show significant improvement or complete resolution with this approach. However, in most cases, especially in intermediate or advanced stages, the primary goal of this approach is to control the infection and reduce mucosal inflammation, thereby creating more favorable conditions for surgical intervention.

Several non-invasive treatments have been proposed to complement antibiotic therapy in MRONJ management. Among the most extensively investigated options are ozone therapy, laser therapy, and hyperbaric therapy. The existing literature does not provide a definitive advantage for these treatments, but they can be considered if available and in consultation with the oncologist [7,18]. Both the Italian and American guidelines suggest incorporating these therapies as adjuncts to other treatments rather than relying on them as primary interventions.

Noteworthy findings have emerged from studies involving teriparatide. Teriparatide is the bioactive component of human parathormone and possesses anabolic properties that promote bone formation [19]. Initial results from animal models showed promising treatment prospects, but clinical use has demonstrated limited efficacy in disease resolution, with sparse literature reporting satisfactory outcomes [20,21]. Additionally, the use of teriparatide has been associated with the potential for new skeletal metastases formation. As a result, its use is contraindicated in oncologic patients, but it remains an intriguing prospect for patients receiving MRONJ-related drugs for the treatment of underlying osteoporosis [22].

#### 3.2.2. Surgical Treatment

The current approach to surgical treatment has undergone significant changes compared to the initial recommendations put forth by the AAOMS. The 2022 update addresses the limitations of previous versions and aligns with the rationale proposed by other international guidelines. This concept was first introduced by the Italian board of the SICMF–SIPMO and has gained traction. Essentially, the surgical approach has evolved from being limited to advanced refractory stages to being considered even in selected Stage 1 MRONJs. According to current literature, MRONJ can be viewed as a focal bone pathology, and adequate removal of the affected bone can lead to disease resolution with an acceptable success rate [23]. Additionally, studies have shown that surgical intervention can positively impact patients’ quality of life and prognosis, with an approximately 10% improvement in survival rate two years after diagnosis [23,24].

The initial notion of sparing already compromised patients (e.g., advanced metastatic malignancies) from potential surgical complications has been surpassed. As a result, both the American and Italian boards recommend patient-specific evaluation of the cost–benefit ratio, without limitations based on the disease stage. 

It is important to note that not all surgical interventions are equal in terms of resolution rates and invasiveness. Procedures such as removal of exposed bony spikes, necrotic bone curettage, or sequestrectomy should not be considered surgical interventions but rather conservative therapies. Surgical interventions are primarily categorized based on the extent of necrotic bone resection. Both the American and Italian guidelines advocate for abandoning the principle of avoiding invasiveness to protect patients’ quality of life and instead prioritize achieving healthy and viable bony margins. Careful planning is crucial and can significantly impact the success of the procedure [23,25]. Most surgeons determine “vital bone” based on intraoperative observation of bleeding margins [7]. However, despite its widespread use, this method lacks definitive evidence of reliability and reproducibility among operators. Therefore, the expertise of the surgeon remains the most critical factor in determining the final outcome. Some authors have explored the use of intraoperative doxycycline and tetracycline fluorescence to assess marginal bone vitality [26,27]. This technique relies on the ability of these molecules to selectively label vital and necrotic bone tissue, allowing the use of a fluorescent lamp to safely identify the vitality of resection margins. Although randomized controlled trials are yet to be conducted, the initial findings and rationale offer interesting perspectives for their systematic application.

Relying solely on bleeding observation to determine the resection margin can be misleading, as macroscopically visible necrotic bone is often associated with varying degrees of surrounding osteomyelitis, potentially influenced by the MRONJ stage [28]. The Italian board recommends the extensive use of MRI due to its superior sensitivity in detecting bone inflammation compared to CT scans [29].

To summarize, surgical treatment aims to achieve safe and healthy margins of the affected bone. This is more straightforward in early MRONJ stages, in which a conservative resection can preserve the anatomical structure. Therefore, the excision of vital margins should be carefully considered, especially in the initial stage, as it is associated with a higher success rate. In advanced MRONJ stages, resection may lead to significant debilitating outcomes (e.g., segmental resection of the mandible), necessitating evaluation for reconstructive procedures. Currently, limited literature exists on flap reconstruction with vascularized bone [30]. Although satisfactory results have been reported, further investigations are needed to address concerns related to prolonged intervention time, hospitalization, and postoperative complications. Alternatively, less invasive reconstructive procedures, although sparsely documented in the literature, can be considered. Reconstruction plates, with or without loco-regional flaps (e.g., pectoralis major myocutaneous flap), remain a reliable approach in managing such defects [31]. Vascularized transplantations offer the advantage of allowing prosthetic dental rehabilitation months after the reconstructive procedure, while avoiding the risk of plate exposure and subsequent surgeries [32]. However, given the short life expectancy of most MRONJ patients, performing extensive reconstructions may be considered overtreatment, favoring a less invasive approach. Further investigations are required to determine the optimal protocol that ensures the best quality of life. It is important to note that quality of life in oncological patients is a multifactorial issue with inherent sources of bias and confounding factors [33].

Another aspect to consider in surgical management is the quality of soft tissue coverage. Previously, MRONJ was viewed as a disease affecting both the bone and mucosal tissue. However, this concept no longer applies to BMA-related MRONJs, in which mucosae and gingiva are involved as a consequence of the underlying affected bone. Conversely, MRONJs resulting from other biologic therapies (e.g., antiangiogenic monoclonal antibodies) present a more complex situation due to the antiangiogenic effect that can impair the mucosal microenvironment. In such cases, the quality of mucosal coverage should be carefully evaluated to avoid potential impairment of the healing process. Recent investigations have shed light on patients with MRONJ who underwent surgical resection of the affected bone, followed by adipose tissue graft application to the surgical site [34]. This procedure aims to harness the beneficial biological effects of adipose-derived stem cells, which play a significant role in modulating the inflammatory response, stimulating the release of various growth factors, and controlling the microenvironment [35].

## 4. Conclusions

Since the initial reports of MRONJ in the early 2000s, our understanding of the pathogenesis, prevention, and treatment strategies has significantly advanced, thanks to the extensive literature published worldwide. National associations dedicated to maxillofacial surgery, oral pathology, and oncology have played a crucial role in developing comprehensive guidelines encompassing all key aspects of this condition. However, certain controversies remain, particularly regarding the standardized use of radiological findings for staging purposes, as proposed in the Italian guidelines. Notably, in its 2022 update, the AAOMS has finally acknowledged the potential for more aggressive surgical treatment even in the early stages of the disease, aligning with the stance advocated by its European counterparts. This agreement supports the hypothesis that radical removal of the affected bone could lead to resolution of MRONJ, contrary to previous suggestions. Nevertheless, the current body of evidence lacks conclusive high-level data, necessitating further studies in the field to provide clarification.

## Figures and Tables

**Table 1:** MRONJ classification based on assumed therapies.

**Bisphosphonate-Related Osteonecrosis of the Jaws (BRONJ)**	Intravenous or oral bisphosphonates
**Non-Bisphosphonate-Related Osteonecrosis of the jaws (non-BRONJ)**	Denosumab (anti-RANKL)
	Bevacizumab, Aflibercept (anti-VEGFR)
	Sunitinib, Sorafenib, Cabozatinib (tyrosine kinase inhibitors)
	Everolimus (anti-mTOR)

**Table 2:** Miller’s classification of tooth mobility. Dental extraction is essential for patients exhibiting Grade 3 mobility in their dental elements. In cases of Grade 2 mobility, dental extraction is recommended but not always obligatory. However, Grade 1 mobility requires individual assessment, considering the urgency to initiate MRONJ-related therapy. It is important to note that patient compliance and financial constraints should be taken into careful consideration when proposing periodontal interventions to preserve the affected teeth.

Grade	Mobility
0	0.2 mm (healthy)
1	0.2–1 mm
2	1–2 mm
3	More than 3–4 mm, with both vertical, vestibolo-lingual, and mesio-distal mobility

**Table 3:** MRONJ-related CT signs acc. with the Italian SICMF–SIPMO guidelines.

Early	Bone cortical erosion
	Thickening of alveolar crest and lamina dura
	Thickening of the trabecular bone
	Focal medullary osteonecrosis
	Persistance of the post-extraction socket
	Widening of the periodontal space
Tardive	Oro-antral, oro-nasal, or oro-cutaneous fistula
	Phatological fracture
	Thickening of the inferior alveolar canal
	Extended osteolysis of the maxillary sinus
	Diffused osteosclerosis of the jaws
	Osteosclerosis of the zygoma and/or hard palate
	Periosteal reaction
	Sinusitis

**Table 4:** Comparison of the Italian SICMF–SIPMO and American AAOMS staging systems.

Stage	SICMF–SIPMO 2020	AAOMS 2022
0	-	Patients with no clinical evidence of necrotic bone but who present with nonspecific symptoms or clinical and radiographic findings (i.e., odontalgia, bone pain, sinus pain, altered neurosensory function, teeth loosening, swelling, alveolar bone loss not related to periodontal disease, osteosclerosis, thickening of the periodontal space)
1	Focal MRONJ: at least one minor clinical sign *; bone addensation observed at the CT scan limited to the alveolar process	Exposed and necrotic bone or fistula that probes to the bone in patients who are asymptomatic and have no evidence of infection/inflammation
2	Diffused MRONJ: at least one minor clinical sign *; bone addensation extended to the basal bone	Exposed and necrotic bone, or fistula that probes to the bone, with evidence of infection/inflammation
3	Severe MRONJ: one or more major ** clinical signs or CT scan demonstrating a mandibular fracture, or an extended osteolysis of the maxillary walls, the zygoma, or the hard palate	Exposed and necrotic bone or fistulae that probes to the bone, with evidence of infection, and one or more of the following: - Exposed necrotic bone extending beyond the region of alveolar bone - Pathologic fracture - Extraoral fistula - Oral–antral/oral–nasal communication - Osteolysis extending to the inferior border of the mandible or sinus floor

According to the SICMF–SIPMO recommendations, all the reported stages should undergo further division depending on the presence of pain (asymptomatic or not) and infection. * Minor clinical signs: halitosis, abscess, mandibular asymmetry, bone exposure, oral fistula(e), mucous flushing, impaired healing following tooth extraction, suddenly developed dental mobility, lip paraesthesia, purulent secretion, spontaneous bone sequestrum, trismus, and soft tissue swelling. ** Major clinical signs: extraoral fistula(e), liquid emission from the nose, abnormal mandibular bone mobility.

## Data Availability

Data available on request to the authors.
